# Temporal Patterns and Predictive Factors of Childhood Depressive Disorders Across Asia

**DOI:** 10.1155/da/5544502

**Published:** 2026-04-29

**Authors:** Kexin Zhang, Chengxia Kan, Yujie Ma, Zhenghui Tian, Fang Han, Xiaodong Sun

**Affiliations:** ^1^ Department of Endocrinology and Metabolism, Affiliated Hospital of Shandong Second Medical University, Weifang, China; ^2^ Department of Endocrinology and Metabolism, Tongji Hospital, School of Medicine, Tongji University, Shanghai, China, tongji.edu.cn; ^3^ Shandong Provincial Key Medical and Health Laboratory for Endocrinology and Metabolic Diseases, Clinical Research Center, Affiliated Hospital of Shandong Second Medical University, Weifang, 261031, China

**Keywords:** bullying victimization, childhood depressive disorders, geographic disparities, temporal trends

## Abstract

**Background:**

Childhood depressive disorders represent a growing mental health concern, yet region‐specific evidence in Asia remains limited. Asia hosts more than half of the global child population and has experienced rapid social and environmental changes that may heighten psychosocial stress. This study assessed the burden, temporal trends, geographic variation, and determinants of childhood depressive disorders across Asia from 1990 to 2023.

**Methods:**

Data for children aged 0–14 years were obtained from the Global Burden of Disease (GBD) 2023 study. Incidence and disability‐adjusted life years (DALYs) were analyzed by age, sex, region, and country. Joinpoint regression quantified annual and long‐term temporal trends. Bullying victimization was evaluated as a behavioral risk factor. Extreme Gradient Boosting (XGBoost) models with Shapley Additive Explanations (SHAP), which show how each variable influences model predictions, were used to identify the major predictors of incidence and DALYs.

**Results:**

In 2023, South Asia showed the highest incidence rate (1099 per 100,000) and DALYs rate (119 per 100,000). Across all regions, children aged 10–14 years and girls had the greatest incidence and DALYs rates. India, China, and Pakistan contributed the largest absolute numbers of cases and DALYs, while Mauritius, Bangladesh, and Pakistan recorded the highest rates. Pakistan demonstrated the steepest long‐term increases in both incidence and DALYs. Bullying‐attributable DALYs increased across all regions, with the largest growth in South and Southeast Asia. SHAP analyses identified age, sex, calendar year, and population size as the strongest predictors, with older children and girls showing markedly higher predicted burdens.

**Conclusion:**

Childhood depressive disorders have increased steadily across Asia over the past three decades, with clear demographic and geographic disparities. These findings highlight the urgency of early detection, school‐based mental health programs, antibullying interventions, and gender‐responsive services. Strengthening child mental health systems in Asia is critical for improving developmental outcomes.

## 1. Introduction

Depressive disorders are a major contributor to the global mental health burden in children and adolescents, affecting emotional development, cognitive function, social engagement, and long‐term well‐being [1, 2]. Early onset depression can have profound and long‐lasting consequences, including impaired academic performance, social dysfunction, and an increased risk of recurrent depressive episodes, self‐harm, and suicide later in life [3, 4]. Despite these serious consequences, extensive research has focused on depression in adults and older adolescents, whereas childhood depression remains relatively underexplored. This imbalance is important because mental health conditions emerging during childhood can shape psychological development and health trajectories across the life course [5, 6].

The urgent need for more research is especially evident in Asia, home to over half of the world’s youth population. Over recent decades, many Asian countries have experienced rapid socioeconomic changes, including urbanization, growing academic pressure, increased digital exposure, and evolving family structures. These shifts have introduced new psychosocial challenges for children [7–14]. At the same time, cultural perceptions of mental health, disparities in healthcare access, and variations in social support systems profoundly influence both the recognition and management of depressive disorders across the region.

@Despite these pressing regional challenges, current epidemiological evidence remains fragmented. While prior Global Burden of Disease (GBD) 2019/2021 studies have extensively reported on depressive disorders and risk‐attributable burdens (e.g., bullying victimization), they typically analyze global aggregates or older demographics, leaving children aged 0–14 largely overlooked [15–17]. Furthermore, independent regional studies are often confined to single countries or limited timeframes [18]. This leaves a critical gap in our understanding of the localized burden, temporal dynamics, and distinct sociodemographic drivers of childhood depression within the rapidly transforming Asian context. Furthermore, the interactive effects of multiple psychosocial stressors on childhood depression remain poorly understood. Traditional epidemiological models and previous GBD studies have predominantly relied on standard stratified analyses, which often assume linear relationships and evaluate risk factors in isolation. This conventional approach fails to capture the complex, nonlinear, and synergistic interactions among various sociodemographic characteristics and psychosocial determinants.

To address these critical gaps, this study presents a comprehensive, updated analysis leveraging the newly released GBD 2023 dataset. To our knowledge, this is the first study to conduct an Asia‐wide analysis specifically assessing the burden and temporal trends (1990–2023) of depressive disorders among children aged 0–14 years, capturing the most recent epidemiological shifts. Beyond simply updating descriptive trends, we introduce a significant methodological advancement to decode these intricate epidemiological dynamics. By employing machine learning algorithms, we aim to provide a granular, quantitative breakdown of how each determinant independently and interactively drives the burden of depressive disorders, including incidence and disability‐adjusted life years (DALYs). Ultimately, this analysis seeks to transcend descriptive trends, offering highly actionable insights to optimize early interventions, guide resource allocation, and inform mental health policymaking for the youngest and most vulnerable populations across Asia.

## 2. Methods

### 2.1. Study Design and Data Source

This population‐based study used estimates from the GBD 2023 study [19, 20]. Data for children aged 0–14 years were extracted for all Asian locations available in the GBD 2023 Results Tool. These locations were grouped into East Asia, South Asia, Southeast Asia, and Central Asia based on the GBD regional classification. The full list of included locations is shown in Table S1.

GBD estimates are model‐derived syntheses of multiple disparate data sources (e.g., systematic reviews, household surveys, and hospital registries) rather than directly observed empirical rates. In this framework, “depressive disorders” were defined using GBD clinical criteria, aligned with the International Classification of Diseases, Tenth Revision (ICD‐10) and the Diagnostic and Statistical Manual of Mental Disorders, Fifth Edition, Text Revision (DSM‐5‐TR). Data included incidence, DALYs, and risk factors, with corresponding 95% uncertainty intervals (UI). DALYs represent the total health loss attributable to a condition and are calculated as the sum of years of life lost due to premature mortality and years lived with disability. For depressive disorders in children, DALYs primarily reflect nonfatal health loss because these conditions contribute mainly through disability rather than direct mortality. The 95% UI indicates the range within which the true value is expected to lie with 95% probability, given uncertainties in data availability, quality, and modeling procedures. Therefore, wider UIs indicate greater uncertainty in the estimates. This study followed the Strengthening the Reporting of Observational Studies in Epidemiology (STROBE) reporting guidelines and was approved by the Institutional Review Board of the Affiliated Hospital of Shandong Second Medical University.

### 2.2. Selection of Study Variables

Age and sex were included as fundamental demographic stratifiers in epidemiological analyses, as they are essential for describing population heterogeneity and disease distribution. Calendar year and location were included to reflect broader temporal and regional differences in mental health awareness, diagnostic practice, and healthcare access across Asia.

### 2.3. Temporal Trend Assessment

We applied joinpoint regression to identify time points at which temporal trends changed significantly and to calculate the annual percent change (APC) for each time segment and the average annual percent change (AAPC) over the full study period. In this context, a joinpoint represents a statistically identified inflection point at which the slope of the trend changes over time. APC describes the annual rate of change within a specific time segment, whereas AAPC summarizes the average rate of change across the entire study period. Positive APC or AAPC values indicate increasing trends, whereas negative values indicate decreasing trends. The model was first constructed in a log‐linear form, and a grid search procedure was then used to identify candidate joinpoints that minimized the mean squared error. Monte Carlo permutation testing was used to determine the most appropriate model, allowing for between zero and five joinpoints. APC was calculated as (e^β − 1) × 100%, where β represents the regression slope. AAPC was obtained by weighting APC values according to the length of each time segment, providing a summary measure of overall trends from 1990 to 2023 [21, 22]. Joinpoint regression identifies temporal changes in trends but does not establish the causes of those changes.

### 2.4. Risk Factor Attribution

Bullying victimization was examined as a GBD‐defined behavioral risk factor for depressive disorders. In the GBD risk hierarchy, risk factors are organized from broader to more specific levels. In our analysis, behavioral risks represented the broad parent category, childhood sexual abuse and bullying represented the intermediate subgroup, and bullying victimization represented the specific exposure. Within the GBD framework, bullying victimization refers to peer bullying among school‐attending children and adolescents, including physical, verbal, relational, and cyberbullying victimization, while excluding abuse by adults, siblings, or intimate partners. Using the GBD comparative risk assessment framework, we extracted depressive disorder DALYs attributable to bullying victimization and summarized regional differences and temporal trends across Asia.

### 2.5. Machine Learning Analysis

We used Extreme Gradient Boosting (XGBoost) to identify predictors of childhood depressive disorder incidence and DALYs across Asia. The analytic unit was the country–year–age–sex stratum. Model inputs included age group, sex, calendar year, and log‐transformed population size, representing the population denominator for each observation. Risk‐attribution variables, such as bullying victimization, were not included because the machine learning analysis focused on core demographic and temporal predictors, whereas bullying victimization was examined separately within the GBD comparative risk assessment framework.

Key hyperparameters, including learning rate, tree depth, subsampling rate, and number of boosting rounds, were optimized using fivefold cross‐validation. Model performance was evaluated using *R* ^2^ and root mean squared error. Shapley Additive Explanations (SHAP) were used to interpret the fitted models. In SHAP summary plots, each point represents one country–year–age–sex observation, and the *x*‐axis reflects the direction and magnitude of each predictor’s contribution to the model output. Positive SHAP values indicate higher predicted incidence or DALYs, whereas negative values indicate lower predicted values. Mean absolute SHAP values were used to rank predictor importance, and dependence plots were used to visualize potential nonlinear effects [23, 24]. To explore possible future trajectories, we generated projections to 2050 by extrapolating from the fitted models based on the observed historical relationships between predictors and outcomes. These forecasts should be interpreted as model‐based extrapolations rather than causal predictions, assuming that the learned relationships remain broadly stable over time and that no major structural changes occur in surveillance, diagnosis, policy, or population health conditions.

### 2.6. Statistical Analysis

Analyses were conducted using R version 4.3.1. Pearson correlation coefficients were calculated to examine associations between predictors and disease outcomes. A *p*‐value < 0.05 was considered statistically significant.

## 3. Results

### 3.1. Burden of Childhood Depression in Asia

**Figure 1:**
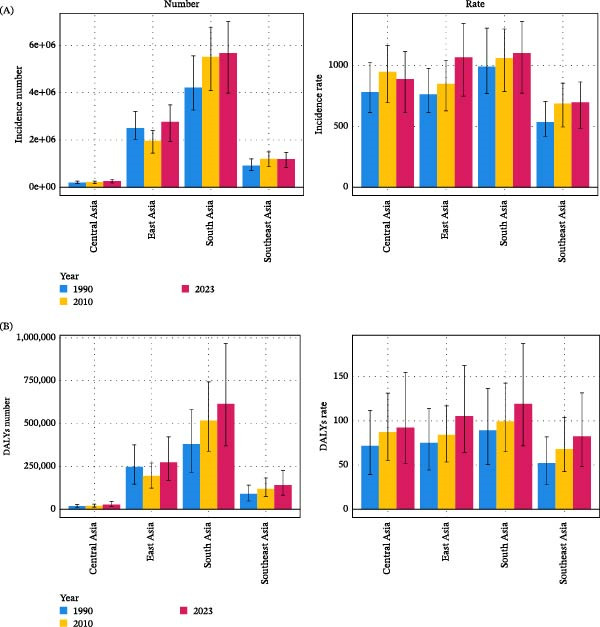
Incidence and DALYs of childhood depressive disorders across Asian regions, 1990–2023. (A) Incidence number and incidence rate. (B) DALYs number and DALYs rate. Rates are expressed per 100,000 population. Asian regions include East Asia, South Asia, Southeast Asia, and Central Asia. DALYs, disability‐adjusted life years. This figure should be interpreted by comparing both absolute burden (numbers) and population‐adjusted burden (rates) across regions and over time.

In 2023, South Asia had the highest incidence burden of childhood depressive disorders and the highest incidence rate, at 1099 per 100,000 (773–1385). Across all four Asian regions, children aged 10–14 years showed the highest incidence counts and rates. In South Asia, this age group had an incidence rate of 2738 per 100,000 (1986–3485). Sex‐specific analyses showed that girls consistently had higher incidence than boys. South Asia had the largest female incidence burden, whereas East Asia reported the highest female incidence rate, at 1401 per 100,000 (973–1789). Over time, South Asia demonstrated the greatest rise in incidence counts, while East Asia showed the largest increase in incidence rates (Figure 1A and Figures S1A,S2A,S3A).

Similarly, South Asia also had the highest DALYs burden and DALYs rate for childhood depression in 2023, at 119 per 100,000 (72–187). Across all four Asian regions, the 10–14‐year age group had the highest DALYs counts and rates. South Asia again showed the greatest burden in this age group, with a DALYs rate of 310 per 100,000 (189–478). Females had higher DALYs numbers and rates than males. South Asia recorded the largest female DALYs burden, with a DALYs rate of 146 per 100,000 (88–226). South Asia demonstrated the greatest increase in DALYs counts over time, while Southeast Asia showed the most pronounced rise in DALYs rates (Figure 1B and Figures S1B,S2B,S3B).

### 3.2. Trends in Incidence and DALY Rates of Childhood Depressive Disorders

**Figure 2:**
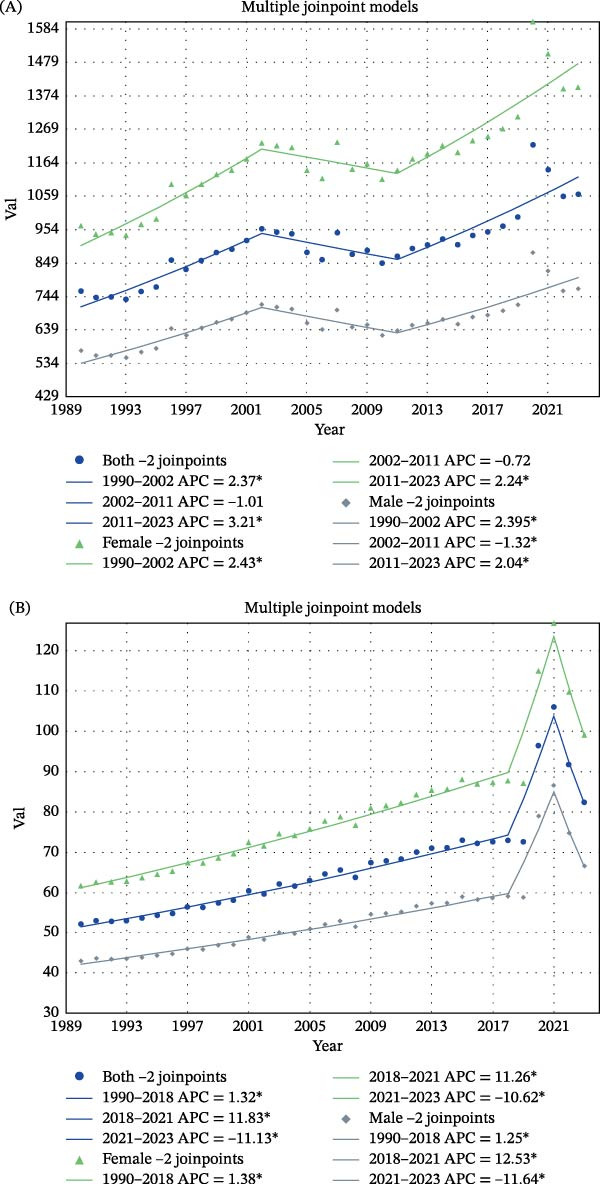
Joinpoint regression analysis of incidence and DALYs rates of childhood depressive disorders in Asia, 1990–2023. (A) Incidence rates. (B) DALY rates. Rates are expressed per 100,000 population. A joinpoint indicates a statistically significant change in the slope of the temporal trend. APC represents the annual percent change within a specific time segment, whereas AAPC represents the average annual percent change across the full study period. Positive APC or AAPC values indicate increasing trends, while negative values indicate decreasing trends. This figure shows temporal changes in rates but does not establish the causes of those changes.

From 1990 to 2023, both the incidence rate and the DALYs rate of depressive disorders among children aged 0–14 years increased substantially, with similar temporal patterns across sexes. In the joinpoint model, each segment represents a period with a distinct annual rate of change, and each joinpoint marks a statistically significant change in that rate. In East Asia, the incidence rate displayed two joinpoints for the combined population. Incidence increased rapidly from 1990 to 2002 (APC 2.37%, *p*  < 0.05), declined slightly between 2002 and 2011 (APC −1.01%, *p*  > 0.05), and then rose again from 2011 to 2023 (APC 2.21%, *p*  < 0.05). Across the full period, the AAPC was 1.38% (95% CI 0.87–1.89, *p*  < 0.001). Girls showed similar but slightly stronger trends, with increases from 1990 to 2002 (APC 2.43%, *p*  < 0.05) and from 2011 to 2023 (APC 2.24%, *p*  < 0.05), resulting in the highest AAPC of 1.49% (95% CI 0.99–2.00, *p*  < 0.001). Boys experienced significant increases from 1990 to 2002 (APC 2.39%, *p*  < 0.05) and from 2011 to 2023 (APC 2.04%, *p*  < 0.05), with an AAPC of 1.24% (95% CI 0.74–1.74, *p*  < 0.001) (Figure 2A).

In Southeast Asia, the DALYs rate also showed two joinpoints. For the combined population, DALYs increased steadily from 1990 to 2018 (APC 1.32%, *p*  < 0.05), followed by a sharp surge between 2018 and 2021 (APC 11.83%, *p*  < 0.05) and a significant decline from 2021 to 2023 (APC −11.13%, *p*  < 0.05). The long‐term AAPC was 1.43% (95% CI 0.97–1.89, *p*  < 0.001). Girls again exhibited the highest burden, with increases from 1990 to 2018 (APC 1.38%, *p*  < 0.05) and from 2018 to 2021 (APC 11.26%, *p*  < 0.05), followed by a decline from 2021 to 2023 (APC −10.62%, *p*  < 0.05). The AAPC reached 1.46% (95% CI 0.99–1.94, *p*  < 0.001). Boys showed similar patterns, with an increase from 1990 to 2018 (APC 1.25%, *p*  < 0.05), a rapid rise from 2018 to 2021 (APC 12.53%, *p*  < 0.05), and a subsequent decline after 2021 (APC −11.64%, *p*  < 0.05), resulting in an AAPC of 1.38% (95% CI 0.92–1.85, *p*  < 0.001) (Figure 2B).

### 3.3. Geographic Distribution

In 2023, India, China, and Pakistan recorded the highest numbers of incident cases of childhood depression. These countries also had the highest DALYs counts.

**Figure 3:**
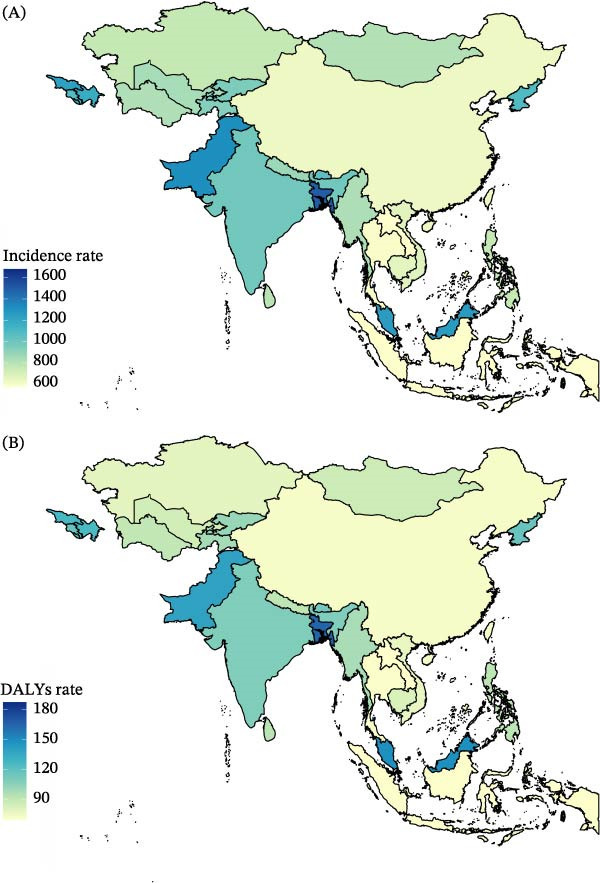
Geographic distribution of childhood depressive disorder burden in Asia, 2023. (A) Incidence rate. (B) DALYs rate. Rates are expressed per 100,000 population. Darker shading indicates a higher burden. This figure should be interpreted as a cross‐sectional comparison of population‐adjusted burden across countries and territories included in the analysis.

Incidence rates ranged from ~576–1680 per 100,000 across Asian countries. The highest rates were observed in Mauritius (1680 per 100,000), Bangladesh (1501 per 100,000), and Pakistan (1318 per 100,000), forming a distinct high‐burden cluster concentrated in South Asia and selected island nations. In contrast, several Southeast Asian countries, including Indonesia and Thailand, recorded the lowest incidence levels (Figure 3A).

A similar pattern appeared for DALYs rates, which ranged from 69 to 188 per 100,000. Mauritius again had the highest rate (188 per 100,000), followed by Bangladesh (165 per 100,000) and Malaysia (149 per 100,000). Pakistan and Georgia also showed elevated DALYs burdens. Countries in mainland Southeast Asia reported comparatively lower DALYs rates, consistent with the incidence distribution. From 1990 to 2023, Malaysia experienced the greatest increase in both the incidence rate and the DALYs rate of childhood depression (Figure 3B).

### 3.4. Risk Factors for DALYs

Across all four Asian regions, the GBD risk framework showed a consistent hierarchical structure for depressive‐disorder DALYs attributable to bullying victimization. Behavioral risks formed the broadest primary category, encompassing lifestyle and psychological behaviors that increase vulnerability to mental disorders. Sexual violence against children and bullying represented a more specific secondary category, reflecting harmful interpersonal experiences associated with later mental health problems. Bullying victimization served as the most specific tertiary factor, representing direct and measurable exposure to repeated peer aggression. This nested structure was observed consistently in East Asia, Central Asia, South Asia, and Southeast Asia.

**Figure 4:**
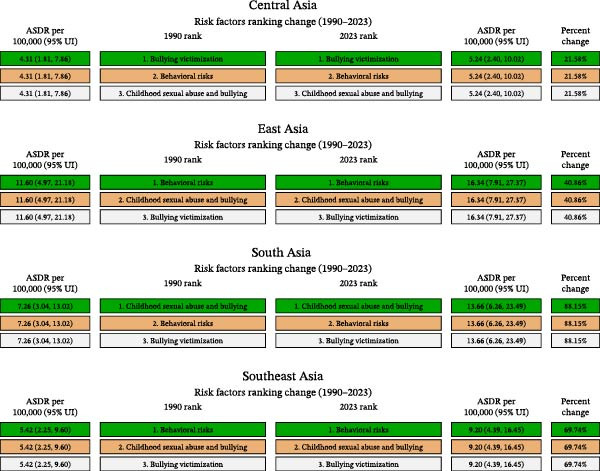
Changes in risk factor rankings for childhood depressive disorder DALYs in Asia, 1990–2023. Depressive‐disorder DALYs attributable to bullying victimization are presented within the GBD risk hierarchy. The hierarchy is shown from broad to specific categories: behavioral risks, childhood sexual abuse and bullying, and bullying victimization. This panel should be interpreted as showing the position of bullying victimization within the GBD risk framework and how its attributable burden changed over time across Asian regions.

For the tertiary factor of bullying victimization, all regions experienced increases from 1990 to 2023, although the magnitude of change varied. In East Asia, the age‐standardized DALYs rate increased from 12 (5–21) to 16 (8–27), a 40.86% increase. Central Asia showed a smaller rise, with rates increasing from 4 (2–8) to 5 (2–10), corresponding to a 21.58% increase. South Asia experienced the largest escalation, with rates rising from 7 (3–13) to 14 (6–23), an 88.15% increase. In Southeast Asia, the rate increased from 5 (2–10) to 9 (4–16), reflecting a 69.74% increase (Figure 4).

### 3.5. SHAP‐Based Machine Learning Interpretation of Incidence and DALYs Rates

**Figure 5:**
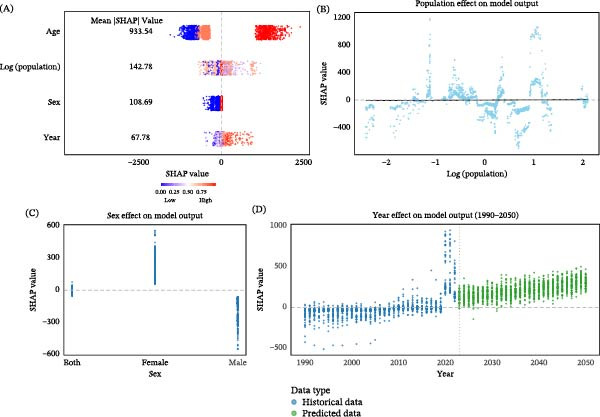
SHAP‐based machine‐learning interpretation of determinants of childhood depressive disorder incidence rates in Asia. (A) SHAP summary plot showing overall predictor importance. (B) SHAP dependence plot for population size. (C) SHAP dependence plot for sex. (D) SHAP dependence plot for calendar year (1990–2050). SHAP, Shapley Additive Explanations. In the summary plot, each point represents one country–year–age–sex observation. The *x*‐axis shows the direction and magnitude of a predictor’s contribution to the model output: positive SHAP values indicate a higher predicted incidence rate, whereas negative values indicate a lower predicted incidence rate. Larger absolute SHAP values indicate stronger effects. Panel D includes model‐based projections to 2050 and should be interpreted cautiously.

The SHAP‐based interpretation of the XGBoost models provided a unified understanding of the key determinants influencing both the incidence and DALYs rates of depressive disorders among children aged 0–14 years across Asia. In Figure 5, positive SHAP values indicate that a given predictor increases the predicted burden, whereas negative values indicate that it reduces the predicted burden; larger absolute SHAP values reflect stronger contributions to the model output. For both outcomes, age emerged as the most influential predictor, consistently showing the highest mean absolute SHAP values (incidence: 934; DALYs: 97). Older children had strong positive SHAP contributions in both models, indicating that the burden of depressive disorders increases markedly from early childhood to adolescence. This pattern likely reflects developmental transitions, rising academic pressure, and increased vulnerability to psychosocial stress during preadolescence and early teenage years.

Population size (log‐transformed) was the second most influential structural factor in both the incidence and DALYs models, although its effect was much smaller than that of age. SHAP patterns suggested a nonlinear association across the observed population range. Intermediate population values tended to show stronger positive contributions, whereas very low or very high values showed more heterogeneous effects. A similar pattern was observed for DALYs, indicating that population context may interact with health system capacity, surveillance intensity, and broader socioeconomic conditions.

Sex exerted consistent effects across the two models. For incidence, girls displayed strong positive SHAP values, indicating higher predicted incidence than boys, who showed predominantly negative contributions. A similar pattern appeared for DALYs, where female sex again contributed positively to the predicted burden. These findings align with epidemiological evidence showing that girls in late childhood and early adolescence have heightened emotional vulnerability, stronger internalizing tendencies, and broader psychosocial risk profiles compared with boys.

Calendar year was a modest but meaningful predictor in both models. For incidence, SHAP values showed a steady upward trend from 1990 to 2023, suggesting that temporal changes and environmental exposures increasingly contributed to rising incidence. A similar pattern was observed for DALYs, where increasing SHAP values indicated a growing disability burden over time. Forecasts for 2023–2050 in both models showed continued upward trajectories, suggesting that without timely intervention, both incidence and DALYs among children may continue to rise in the coming decades (Figure 5 and Figure S4).

## 4. Discussion

This study provides a comprehensive assessment of childhood depressive disorders across Asia from 1990 to 2023, revealing substantial and increasing burdens with pronounced geographic, age‐related, and sex‐specific differences. South Asia consistently demonstrated the largest absolute burden and high rates of childhood depressive disorders, reflecting both its population structure and increasing psychosocial pressures experienced by children. India, China, and Pakistan contributed the greatest absolute numbers of cases and DALYs, aligning with their dominant share of the regional child population and underscoring their central role in the overall childhood mental health landscape in Asia.

Children aged 10–14 years showed the highest incidence and DALYs rates across all regions. This developmental stage is characterized by rapid physical, emotional, and social changes. Academic pressure intensifies, peer comparison becomes more prominent, and engagement with digital media increases, all of which heighten vulnerability to emotional difficulties. The transition from late childhood to early adolescence also brings greater sensitivity to stress and evolving expectations within family and school environments. These findings underscore the need to strengthen school‐based mental health support, as schools represent the primary setting where children spend most of their daily lives [25]. Collectively, the evidence identifies late childhood and early adolescence as a critical window for targeted mental health interventions.

Sex differences were notable and consistent. Girls had higher incidence and disability rates in almost all regions. Biological factors, including earlier puberty and greater hormonal sensitivity, may play a role. Girls also tend to show more internalizing behaviors and may be more affected by interpersonal stress. In addition, girls in several Asian cultures face strong expectations related to academic performance, behavior, and social appearance. These pressures can contribute to emotional strain. The high burden among girls emphasizes the need for gender responsive prevention and support programs [26].

Temporal trends showed sustained increases across most Asian regions from 1990 to 2023. Some regions experienced periods of accelerated growth. The sharp rise in disability rates between 2018 and 2021 in Southeast Asia coincided with the COVID‐19 pandemic period and may be associated with multiple concurrent social and environmental disruptions [1, 27]. During this period, children experienced school closures, reduced interaction with friends, increased family tension, and greater screen exposure [1, 28–30]. These disruptions may have intensified emotional distress [31]. However, joinpoint analysis identifies changes in temporal trends rather than their causes, and these inflection points should therefore be interpreted cautiously rather than as direct evidence of causal effects. Although disability rates declined after 2021, they remained above prepandemic levels, suggesting that recovery may be gradual and that some children may continue to experience lingering psychosocial effects.

Geographic differences were substantial. South Asia showed both large numbers and high rates of childhood depression. Mauritius, Bangladesh, and Pakistan recorded some of the highest incidence and disability rates. These patterns may reflect a combination of demographic density, social stress, limited mental health resources, and slower recognition of childhood mental health conditions. In contrast, countries in mainland Southeast Asia generally had lower incidence and disability rates. These differences may relate to variations in health system capacity, family structures, cultural norms, and differences in how childhood mental health issues are recognized or reported. Pakistan showed the largest long‐term increases in both incidence and disability rates. Political instability, economic stress, and limited access to mental health services may have contributed to this rise [32].

Bullying victimization emerged as a strong contributor to disability burden in all regions. The burden attributable to bullying increased steadily from 1990 to 2023, with the largest increases in South Asia and Southeast Asia. Bullying is a known risk factor for emotional disorders. Repeated peer aggression can lead to fear, social withdrawal, low self‐esteem, and long‐term depressive symptoms. The rising levels of bullying victimization suggest that interpersonal and school‐related stress has intensified for children in recent decades [33]. These findings support the need for stronger school policies, teacher training, and child protection measures.

This study focuses specifically on children aged 0–14 years in Asia, a demographic and regional context that has been underexamined in previous global assessments. By narrowing the scope to early life, we identified substantial regional heterogeneity, with marked burdens in South and East Asia. Furthermore, by integrating XGBoost and SHAP, we moved beyond conventional descriptive analyses to better characterize the complex determinants of this burden. This machine‐learning framework confirmed that age is the strongest predictor of both incidence and DALYs, reinforcing the vulnerability of older children. Female sex increased predicted burden in both models, which supports observed epidemiological patterns. Population size also affected predictions. Medium‐sized populations showed the strongest positive contributions, suggesting that demographic pressure interacts with health system readiness and social conditions. Calendar year had a steady upward effect in both models. This shows that the overall environment for child mental health in Asia has become increasingly challenging. The projected increase to 2050 highlights the risk of continued growth if no major intervention is made.

This study has several limitations. First, all estimates were derived from the GBD 2023 framework and therefore depend on the availability and quality of underlying data sources as well as statistical modeling procedures. Second, the joinpoint and machine learning analyses were intended to identify temporal patterns and predictive factors rather than establish causal relationships. In addition, although the full 0–14‐year age range was included in accordance with the GBD childhood classification, estimates for children aged 0–4 years should be interpreted cautiously because case ascertainment is more difficult in this age group and relies more heavily on limited data and model‐based estimation.

Taken together, these findings highlight the need to strengthen child and adolescent mental health systems in Asia. The observed age and sex disparities, along with the contribution of bullying victimization, support several evidence‐based priorities, including earlier identification during late childhood, stronger school‐based mental health support, expanded community services, and targeted antibullying strategies. Given the consistently higher burden among girls, prevention and care models should also be gender responsive. Broader approaches, such as strengthening national policy support, expanding the mental health workforce, and exploring culturally appropriate digital tools, may serve as valuable complements to these efforts and help improve long‐term resilience.

## 5. Conclusion

Childhood depressive disorders remain a major and increasing public health concern across Asia. South Asia bore the highest absolute burden, whereas children aged 10–14 years and girls consistently showed the highest incidence and DALY rates. Several countries, including Pakistan, India, China, and Bangladesh, experienced high or rising burdens. Bullying victimization contributed substantially to depressive‐disorder DALYs across all regions, with particularly marked increases in South Asia and Southeast Asia. Machine learning analyses further identified age, sex, calendar year, and population size as important predictors of incidence and DALYs. Overall, these findings indicate substantial and uneven childhood depressive burden across Asia and underscore the importance of timely, context‐sensitive prevention and care.

## Author Contributions

Kexin Zhang, Chengxia Kan, Zhenghui Tian, and Yujie Ma collected, analyzed, and interpreted the data. Chengxia Kan and Kexin Zhang drafted the manuscript.

## Funding

This study was funded by grants from the Special Funds for the Taishan Scholars Program of Shandong Province (Grant tsqn202211365) and Bill and Melinda Gates Foundation.

## Disclosure

All the authors conceived and designed the study, and revised and approved the final version.

## Conflicts of Interest

The authors declare no conflicts of interest.

## Supporting Information

Additional supporting information can be found online in the Supporting Information section.

## Supporting information


**Supporting Information** Figure S1: Age‐group contributions to incidence and DALYs of childhood depressive disorders in four Asian regions, 1990 and 2023. (A) Age‐group distribution of incidence number and incidence rate. (B) Age‐group distribution of DALYs number and DALYs rate. Rates are expressed per 100,000 population. DALYs, disability‐adjusted life years. This figure should be interpreted by comparing age‐specific contributions to both absolute and population‐adjusted burden. Figure S2: Temporal trends in incidence and DALYs of childhood depressive disorders across Asian regions, 1990–2023. (A) Trends in incidence number and DALYs number. (B) Trends in incidence rate and DALYs rate. Rates are expressed per 100,000 population. This figure should be interpreted as showing regional variation in long‐term burden trends. Figure S3: Sex differences in incidence and DALYs of childhood depressive disorders across Asian regions, 2023. (A) Incidence number and incidence rate. (B) DALYs number and DALYs rate. Rates are expressed per 100,000 population. This figure should be interpreted by comparing male and female burden in both absolute and population‐adjusted terms. Figure S4: SHAP‐based machine‐learning interpretation of determinants of childhood depressive disorder DALYs rates in Asia. (A) SHAP summary plot showing overall predictor importance. (B) SHAP dependence plot for population size. (C) SHAP dependence plot for sex. (D) SHAP dependence plot for calendar year (1990–2050). SHAP, Shapley Additive Explanations. Positive SHAP values indicate that a predictor increases the predicted DALYs rate, whereas negative values indicate that it lowers the predicted DALYs rate. Panel D includes model‐based extrapolations and should be interpreted cautiously.

## Data Availability

The data that support the findings of this study are available from the GBD 2023 at https://ghdx.healthdata.org/gbd-2023.
