# Supplementary material for: Temporal Patterns and Predictive Factors of Childhood Depressive Disorders Across Asia
Source: Depress Anxiety. 2026 Apr 29;2026:5544502. doi: 10.1155/da/5544502 (PMC13129407; doi:10.1155/da/5544502)
Supplement: Supplementary file 1 — Supporting Information Figure S1: Age‐group contributions to incidence and DALYs of childhood depressive disorders in four Asian regions, 1990 and 2023. (A) Age‐group distribution of incidence number and incidence rate. (B) Age‐group distribution of DALYs number and DALYs rate. Rates are expressed per 100,000 population. DALYs, disability‐adjusted life years. This figure should be interpreted by comparing age‐specific contributions to both absolute and population‐adjusted burden. Figure S2: Temporal trends in incidence and DALYs of childhood depressive disorders across Asian regions, 1990–2023. (A) Trends in incidence number and DALYs number. (B) Trends in incidence rate and DALYs rate. Rates are expressed per 100,000 population. This figure should be interpreted as showing regional variation in long‐term burden trends. Figure S3: Sex differences in incidence and DALYs of childhood depressive disorders across Asian regions, 2023. (A) Incidence number and incidence rate. (B) DALYs number and DALYs rate. Rates are expressed per 100,000 population. This figure should be interpreted by comparing male and female burden in both absolute and population‐adjusted terms. Figure S4: SHAP‐based machine‐learning interpretation of determinants of childhood depressive disorder DALYs rates in Asia. (A) SHAP summary plot showing overall predictor importance. (B) SHAP dependence plot for population size. (C) SHAP dependence plot for sex. (D) SHAP dependence plot for calendar year (1990–2050). SHAP, Shapley Additive Explanations. Positive SHAP values indicate that a predictor increases the predicted DALYs rate, whereas negative values indicate that it lowers the predicted DALYs rate. Panel D includes model‐based extrapolations and should be interpreted cautiously. [file DA-2026-5544502-s001.docx]

**Supplementary material**

**Supplementary Table S1.** Included Asian locations and regional classification used in this study

| **Asian subregion** | **Locations included** |
| --- | --- |
| East Asia | China; Democratic People’s Republic of Korea; Taiwan |
| South Asia | Bangladesh; Bhutan; India; Nepal; Pakistan |
| Southeast Asia | Cambodia; Indonesia; Lao People’s Democratic Republic; Malaysia; Maldives; Mauritius; Myanmar; Philippines; Seychelles; Sri Lanka; Thailand; Timor-Leste; Viet Nam |
| Central Asia | Armenia; Azerbaijan; Georgia; Kazakhstan; Kyrgyzstan; Mongolia; Tajikistan; Turkmenistan; Uzbekistan |

**Supplementary Figures**


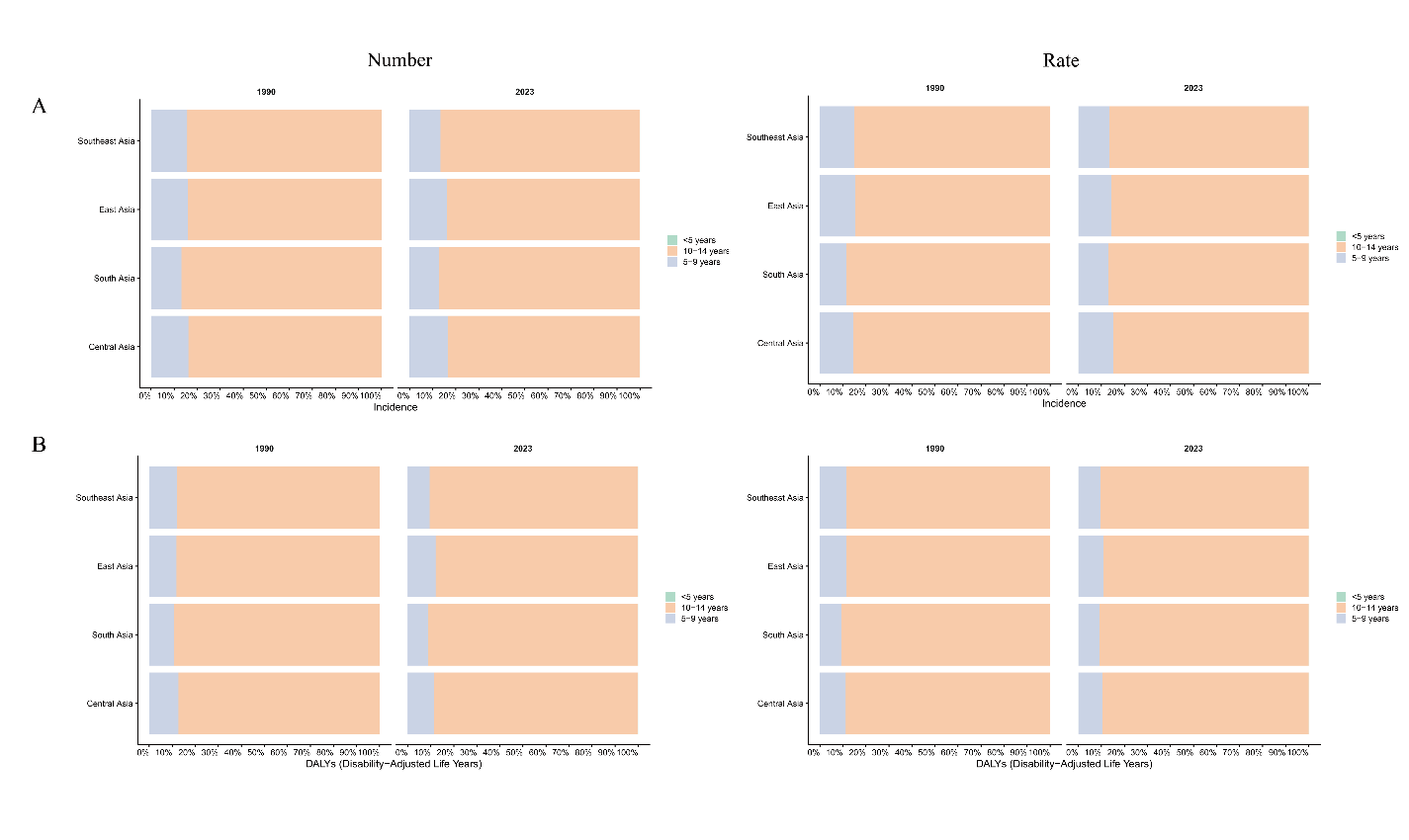


**Supplementary Figure 1. Age-group contributions to incidence and DALYs of childhood depressive disorders in four Asian regions, 1990 and 2023.** (A) Age-group distribution of incidence number and incidence rate. (B) Age-group distribution of DALYs number and DALYs rate. Rates are expressed per 100,000 population. DALYs, disability-adjusted life years. This figure should be interpreted by comparing age-specific contributions to both absolute and population-adjusted burden.


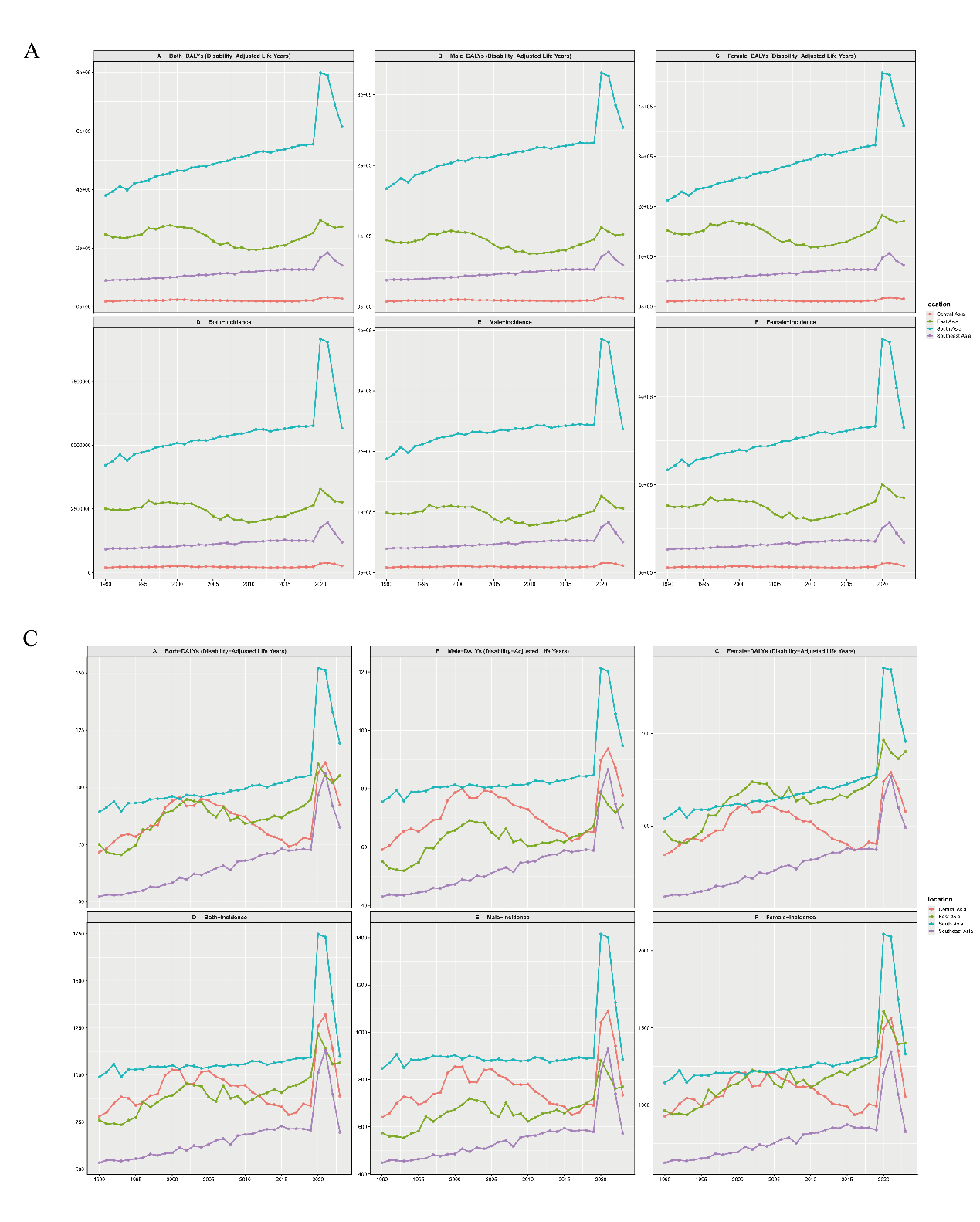


**Supplementary Figure 2. Temporal trends in incidence and DALYs of childhood depressive disorders across Asian regions, 1990–2023.**(A) Trends in incidence number and DALYs number. (B) Trends in incidence rate and DALYs rate. Rates are expressed per 100,000 population. DALYs, disability-adjusted life years. This figure should be interpreted as showing regional variation in long-term burden trends.


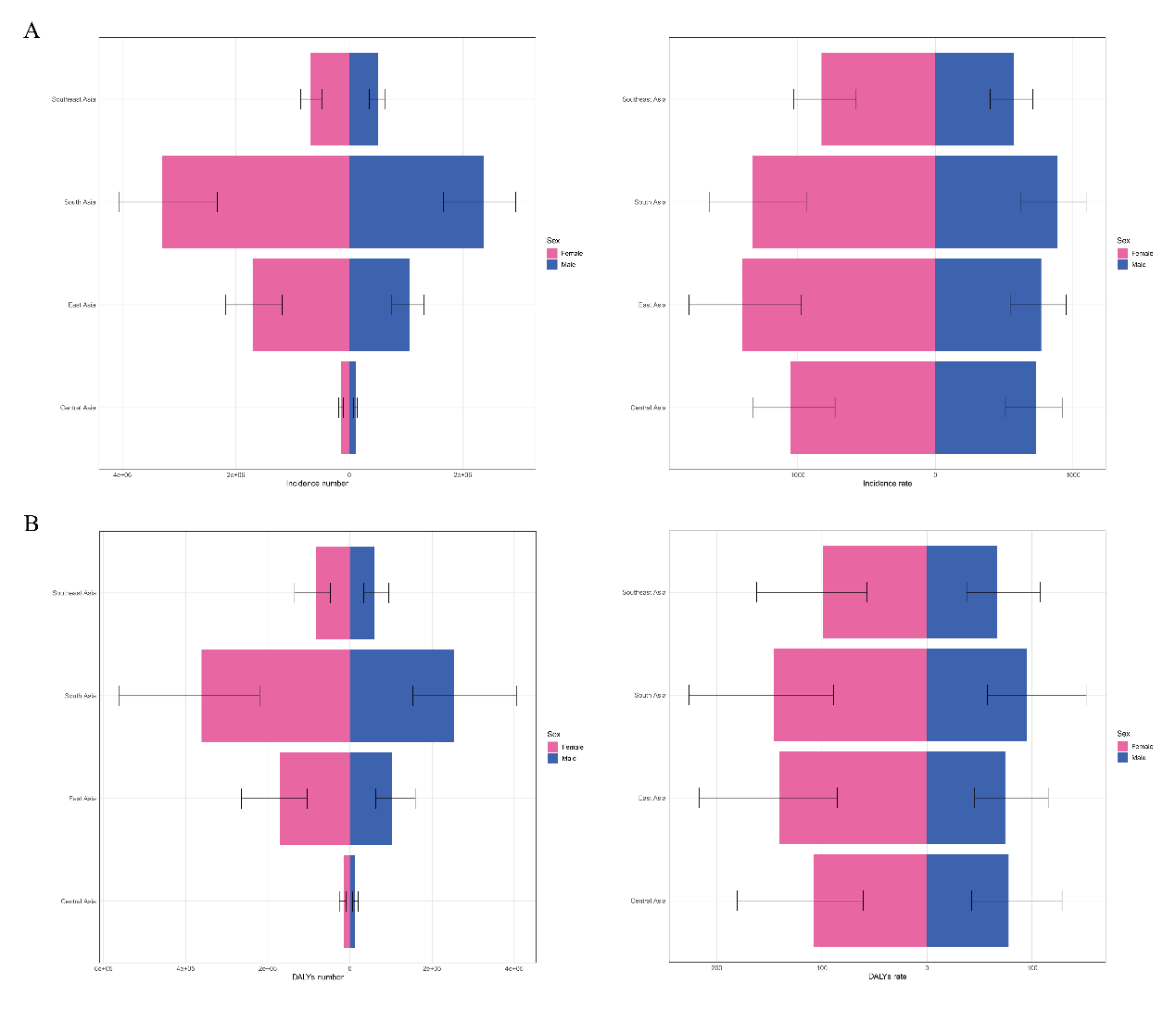


**Supplementary Figure 3. Sex differences in incidence and DALYs of childhood depressive disorders across Asian regions, 2023.** (A) Incidence number and incidence rate. (B) DALYs number and DALYs rate. Rates are expressed per 100,000 population. DALYs, disability-adjusted life years. This figure should be interpreted by comparing male and female burden in both absolute and population-adjusted terms.


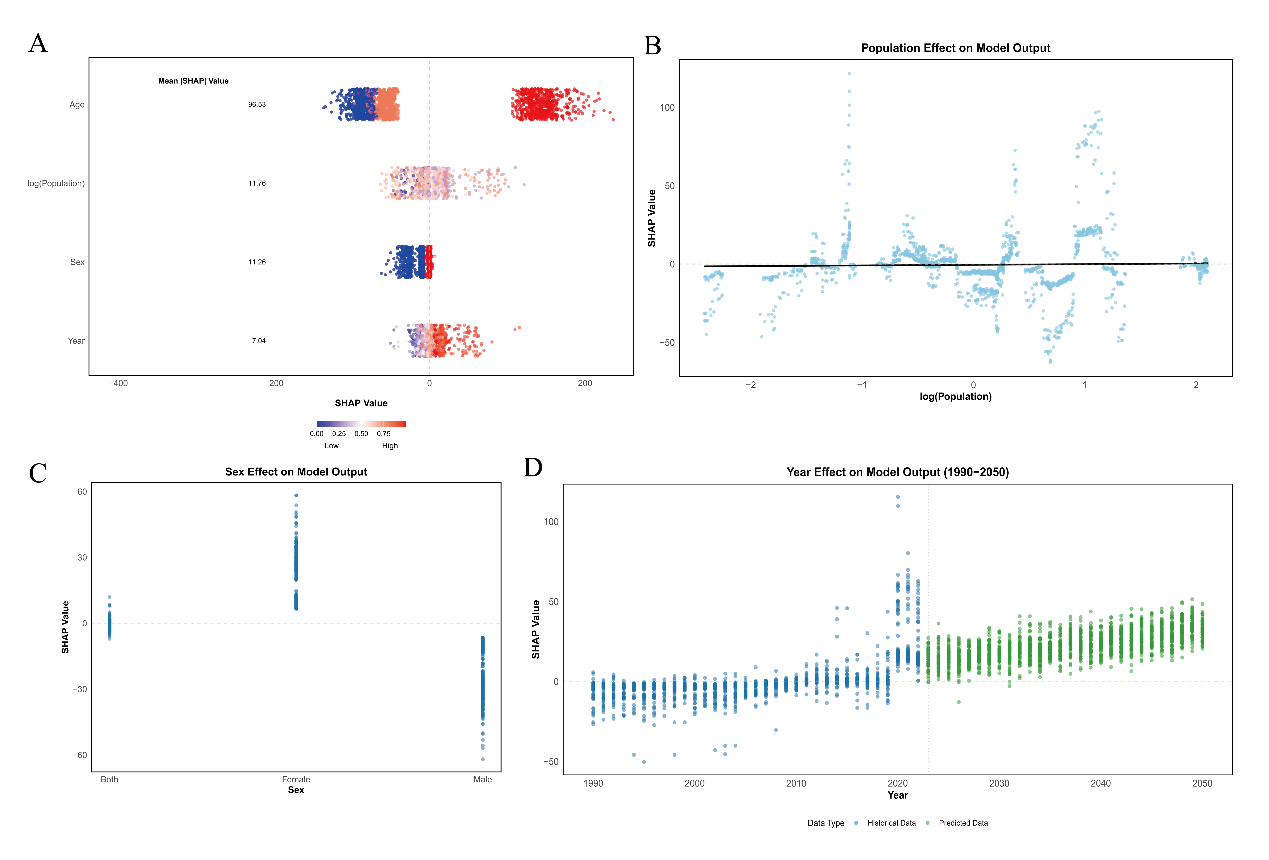


**Supplementary Figure 4. SHAP-based machine-learning interpretation of determinants of childhood depressive disorder DALYs rates in Asia.** (A) SHAP summary plot showing overall predictor importance. (B) SHAP dependence plot for population size. (C) SHAP dependence plot for sex. (D) SHAP dependence plot for calendar year (1990–2050). SHAP, Shapley Additive Explanations; DALYs, disability-adjusted life years. Positive SHAP values indicate that a predictor increases the predicted DALYs rate, whereas negative values indicate that it lowers the predicted DALYs rate. Panel D includes model-based extrapolations and should be interpreted cautiously.
